# Retraction Note: Dietary polyphenols and the risk of metabolic syndrome: a systematic review and meta-analysis

**DOI:** 10.1186/s12902-024-01810-2

**Published:** 2024-12-12

**Authors:** Pushpamala Ramaiah, Kamilya Jamel Baljon, Ahmed Hjazi, Maytham T. Qasim, Omar Abdulwahid Salih Al-ani, Shad Imad, Beneen M. Hussien, Ali Alsalamy, Nazila Garousi

**Affiliations:** 1 Faculty of Nursing, Umm al- Qura University, Makkah, Saudi Arabia; 2 Department of Medical Laboratory Sciences, College of Applied Medical Sciences, Prince Sattam bin Abdulaziz University, Al-Kharj, 11942 Saudi Arabia; 3 Department of Anesthesia, College of Health and Medical Technololgy, Al-Ayen University, Thi-Qar, Iraq; 4 Department of Pharmacy, Al Rafidain University College, Bagdad, Iraq; 5 Medical Technical College, Al-Farahidi University, Baghdad, Iraq; 6 Medical Laboratory Technology Department, College of Medical Technology, The Islamic University, Najaf, Iraq; 7 College of medical technology, Imam Ja’afar Al-Sadiq University, Al‐Muthanna, 66002 Iraq; 8 Department of Clinical Nutrition, School of Nutrition and Food Science, Isfahan University of Medical Sciences, Isfahan, Iran

**Retraction Note: BMC Endocrine Disorders (2024) 24:26**

10.1186/s12902-024-01556-x

The Editors have retracted this article after an investigation by the Publisher found irregularities with respect to submission and authorship. Pushpamala Ramaiah and Kamilya Jamel Baljon have stated that they had no prior knowledge of this article. The Editors have been unable to establish the current email addresses for Shad Imad, Ali Alsalamy and Ahmed Hjazi. Pushpamala Ramaiah and Kamilya Jamel Baljon disagree to this retraction. P Maytham T. Qasim, Omar Abdulwahid Salih Al-ani, Beneen M. Hussien and Nazila Garousi did not respond to correspondence from the Editors about this retraction.

